# Supplementary material for: Are carbon emissions trading and green financial instruments synergistic? -Comprehensive quantitative research based on content analysis
Source: PLoS One. 2024 Mar 7;19(3):e0298601. doi: 10.1371/journal.pone.0298601 (PMC10919601; doi:10.1371/journal.pone.0298601)
Supplement: S1 Data — (ZIP) [file pone.0298601.s001.zip › Data/地级市Z_F工作报告.zip!/║┌┴·╜¡/╞▀╠¿║╙╩╨/└┤╫╘╣1⁄2╓┌║┼í╛┬φ┐╦╩2╛▌═°í┐_2016.docx]

各位代表：

　　现在，我代表市人民政府，向大会报告工作，请予审议，并请政协委员和列席人员提出意见。

　　一、2015年工作回顾

　　2015 年是“十二五”收官之年，也是我市转型发展攻坚克难、砥砺奋进的一年。一年来，面对煤焦市场持续低迷的严峻形势，我们迎难而上，知难而进，在省委省政府和市委正确领导下，在市人大和市政协大力支持下，全力以赴稳增长、促改革、调结构、惠民生，经济实现逐步企稳，社会保持和谐稳定。预计全市生产总值实现220 亿元，比上年增长4.0%；规模以上工业增加值实现53 亿元，增长3.0%；财政一般公共预算收入实现17.2 亿元，下降4.2%，其中市本级实现11.9 亿元，增长12.4%；城镇居民人均可支配收入实现20790 元，增长3.6%；农村居民人均可支配收入实现10996元，增长9.0%。

　　产业升级步伐加快。加快改造提升传统产业，多元发展替代产业，积极培育新兴产业，全年推进重点产业项目50 个。全市产业项目投资完成56.2 亿元，占固定资产投资比重58.0%，提高7.0 个百分点。全省在建最大煤化工项目宝泰隆30 万吨稳定轻烃、投资25 亿元的隆鹏清洁化学品项目进展顺利，煤炭循环经济链条进一步延伸。奥瑞德蓝宝石二期、宝泰隆石墨烯项目开工建设，华夏锂离子电池完成主体工程，新材料产业实现新的突破。万通管业、隆发生物质油、双叶公司拼板弯木车间等非煤项目建成投产，勃农兴达玉米脱粒机项目产值超过1亿元。粮食生产实现十二连增，产量达24 亿斤。全市绿色食品种植面积90万亩，获得有机食品标识17个，地理标识认证4个，金瀛纳豆、乐宝赤豆沙、天泰鲜玉米等深加工项目投产。第三产业快速发展，中国供销商贸博览城开工建设，七台河火车站集装箱中转物流、弘信物流东方建材城建成运营，泛东北亚大宗商品国际交易中心上线试运行；制定专项扶持政策，大力推动电子商务发展，建成7 家市、县区级电子商务产业园，70余户知名电商和本地网商入驻园区，实现交易额2.0 亿元，带动创业就业1900 余人，勃利县被评为国家电子商务进农村综合示范县。产业集聚承载能力增强，七台河煤炭矿区总体规划获国家发改委批复，再生资源回收利用园区主体工程基本完成，经济开发区、金沙健康产业园、新兴木制品园区基础设施进一步完善，勃利老工业区搬迁改造获国家支持7684 万元。招商引资取得新成效，引进落地项目20个，总投资81亿元。

　　民生保障持续改善。坚持经济困难时期民生投入力度不减，财政民生支出占总支出比重达73.1%，比上年提高2.8个百分点。保障性安居工程深入推进，煤矿棚户区完成安置分配8605套，采煤沉陷区棚户区改造工程开工建设4421 套；建成城市棚改房800 套、公租房273 套，改造农村泥草房危房476 户，完成暖房子工程100 万平方米。社会保障能力显著提升，新农保和城居保统一合并为居民养老保险，城镇居民基本医疗保险财政补助标准提高到380元，城市低保标准提高到每月500元，农村低保标准提高到每年3500 元。出台加快发展养老服务业实施意见，市失能失智老人养护院完成主体工程，社会福利院改扩建工程开工建设，扶持和规范民办养老机构发展，民办养老机构运营补贴提高到每床每月200 元。教育事业加快发展，市职业技师学院实训基地、市一中信息综合楼、市实验幼儿园等投入使用，桃山区、新兴区通过国家义务教育均衡发展评估检查。医疗卫生水平进一步提升，市人民医院外科楼基本建成，市妇幼保健院、市县急救中心投入使用，医联体实现城乡全覆盖，完善了专家出诊、分级诊疗和双向转诊机制，为乡镇卫生院招聘医科大学生18名。文体事业繁荣发展，市体育场建成使用，建成社区文化中心25个、文化活动室12个。大力推动创业就业，为643人发放小额贷款3215万元，新增就业7400人；积极帮助七矿公司解困发展，落实帮扶资金2.2亿元，已安置和正在落实安置富余人员2348人。认真解决涉及群众利益的热点难点问题，新建改扩建福泽、安民等社区服务用房6处；建成市公交调度指挥中心、北岸新城和金沙新区停车场等3 处公交场站，更新新型环保公交车50 台；办结拖欠农民工工资案件64 起，解决拖欠工资3779 万元；“民声之窗”受理解决群众诉求160件次。

　　城乡建设扎实推进。城市供水隧洞工程稳步推进，泵站工程开工建设，管线工程完成前期工作。城乡路网骨架进一步完善，西环路开工建设，七桦路、松涛路改扩建工程和碾子河大桥新建工程竣工通车，七桃路、湖东路、幸福路维修改造工程如期完工，建成农村公路148.6 公里。完善“三供三治”基础设施，新建改造供热管网38.6 公里，新建开发区至金沙新区供热、供水主管网，如意家园、福泽家园换热站建成运行，福泽家园、林苑之星天然气储配站建设完成；锦江集团垃圾发电项目开工建设，如意家园垃圾中转站投入使用，第二污水厂主体工程及污水截流管网工程完工试运行。实施绿化景观升级改造，新增建成区绿地面积10.5 公顷。加强大气污染治理，淘汰老旧黄标车1885 辆，淘汰改造小锅炉65 台，完成宝泰隆公司等3 家电厂除尘治理和脱硝脱硫升级改造；新建3 座空气质量自动监测站，空气质量达标天数320 天。加强水源保护，建成松花江水污染防治项目11 个，清理违法生产企业12 家。进一步完善城市管理“六化一考”制度，文明城市建设管理品位和水准进一步提升，全省文明城市建设现场会在我市召开。

　　重点改革不断深化。加快行政审批制度改革，保留行政审批事项195 项。实行产业项目并联审批，减少审批流程80个，流程优化率24.8%；减少审批前置件172 个，占总审批事项比重为28.2%；单项审批时限最多压缩70 天，提速率平均达到42.2%。建立权力清单、责任清单制度，进一步厘清部门职责，市本级行政权力减少40%。政府机构改革全面完成，事业单位分类改革稳步推进。落实商事制度改革，降低准入门槛，实施“四证合一”、“一照一码”登记制度，新增市场主体3461 户。完善政府收支预算、部门预算改革，推行公务卡制度改革，完成区级财政国库集中支付转轨工作。推进投融资体制改革，采取PPP 模式实施信息惠民工程，燃气供应、垃圾污水处理等公共服务项目建设引入民营资本。积极推进农业综合改革，扎实推进农村土地承包经营权确权登记试点、农村集体产权制度改革试点，加强农民合作社规范化标准化建设，市级规范社达到99 个。深化医疗卫生体制改革，破除“以药补医”，市直3 所公立医院探索推行“两取消、两降低、六提高”综合改革措施。改革城乡困难群众重特大疾病医疗救助机制，新增3家即时结算医院。

　　社会保持和谐稳定。严格落实党政同责和属地化管理责任，煤矿、危险化学品、消防、食品药品、道路交通等各行业各领域安全生产形势保持稳定。加强煤矿安全分类监管，落实24 小时旁站式监管制度，完成全市煤矿水患物探工作，采取断电和火工品专项整治行动，严厉打击煤矿违法非法开采，111 处矿井通过省整治整合批复，落实关闭矿井任务51处。全面加强社会管理综合治理，保持对违法犯罪的高压态势，社会治安形势稳定，成功破获公安部重点督办的“9·8”特大系列地下钱庄案。推进食品药品“四有两责”落实，实现食药监管网络乡镇村屯全覆盖。实行属事属地联合化解、疑难信访问题综合评议、市级领导信访接待日等工作制度，开展“化积案、降访量”专项行动，化解了一批信访疑难案件，进京非访总量下降32.6%。

　　政府建设不断加强。扎实开展“三严三实”专题教育，针对“庸懒散浮”、“不作为、慢作为、乱作为”、“四风”等问题进行专项整治，整改问题236 个。开展廉洁自律专项检查，全年查处违纪案件337 件。严控“三公”经费支出，全市“三公”经费下降5.2%。加强财政支出监管和审计监督，政府投资评审项目支出审减额3 亿元，市本级政府采购节支2147 万元，审计查出违规资金7274 万元。强化和完善市政务服务中心、企业投诉服务中心职能，网上政务服务中心开通试运行。严格执行市人大及其常委会的各项决议决定，自觉接受市人大及其常委会的法律监督、工作监督和市政协的民主监督，办理人大代表建议88 件、政协委员提案167 件，代表、委员满意率达98%。

　　各位代表，过去一年，在艰难形势下我市经济社会平稳发展，成绩来之不易。这是省委省政府和市委统揽全局、正确领导的结果，是市人大、市政协有力监督、大力支持的结果，是各级各部门和全市人民团结拼搏、共同奋斗的结果。在此，我谨代表市人民政府，向全市广大干部群众，向关心支持我市发展建设的各界友人，表示衷心的感谢和崇高的敬意！

　　在肯定成绩的同时，我们也要清醒地认识到，我市经济社会发展以及政府自身建设仍存在着一些亟待解决的问题：结构性矛盾突出，煤焦主导产业抵御市场风险能力不强，非煤替代产业对经济发展尚未形成有力支撑，稳增长的基础薄弱；整体经济形势短时间难以改善，保运转、保民生支出压力增大，就业、信访、环保、社会稳定面临诸多挑战；公共安全领域基础薄弱，煤矿和危化等领域本质安全水平亟待提升；一些干部担当意识不强，抓发展、破难题的工作能力和水平不高，少数政府工作人员不作为、慢作为、乱作为，群众和社会反响较大等，这些问题，我们一定高度重视，并切实加以解决。

　　二、2016年工作安排

　　2016 年是实施“十三五”规划、全面建成小康社会决胜阶段的开局之年，也是经济结构性改革的攻坚之年。在经济新常态的能源需求格局下，煤炭产能过剩形势短时间内不会改变，结构不优、总量不足、质量不高、活力不强仍然是我市经济发展的主要矛盾，去产能、去库存、去杠杆、降成本、补短板的难度和压力很大。但我们更要看到积极变化和有利因素。我国经济保持“四个没有变”的良好态势，潜力大、韧性好，大众创业、万众创新蓬勃兴起，新技术、新产业、新业态、新模式不断涌现；国家加快东北振兴和促进资源型城市可持续发展，推动“互联网+”、中国制造2025、加大供给侧结构性改革力度等系列政策措施，以及我省推进“龙江陆海丝路带”建设、深入实施“五大规划”，特别是我市纳入《长白山林区生态保护和经济转型发展规划》，这些都将为我市转型发展提供新的发展动力和政策机遇。我们有“十二五”打下的坚实基础，一批项目开始发力见效；不断深化重点领域改革，发展活力将进一步释放；困难时期全市上下人心思变、人心思进，干事创业氛围更加浓厚。只要我们保持定力，抢抓机遇，爬坡过坎，滚石上山，就一定能够战胜困难，不断取得各项事业新胜利。2016年政府工作的总体要求是：以党的十八大、十八届三中、四中、五中全会精神为指导，按照中央、省委经济工作会议和省委十一届六次全会、市委八届六次全会的部署，牢固树立“五大发展”理念，深入贯彻落实“四个全面”战略布局，坚持依托煤、延伸煤、不唯煤、超越煤发展思路不动摇，以促进经济转型发展为主线，以增强可持续发展能力为核心，加大结构性改革力度，大力优化经济发展环境，强力推进招商引资上项目，切实保障和改善民生，全力维护安全稳定，不断开创经济社会转型发展新局面。

　　发展主要预期目标是：地区生产总值增长6.5%以上；规模以上工业增加值增长3%以上；财政一般公共预算收入增长5%以上；固定资产投资增长7%以上；社会消费品零售总额增长6%以上；城镇和农村居民人均可支配收入分别增长5%和7.8%。

　　全年重点抓好八个方面工作。

　　（一）大力推进产业项目建设，强化经济转型发展基础

　　重点推进五千万元以上产业项目32 个，总投资202 亿元，年度投资57.4 亿元，其中亿元以上项目23 个。大力发展非煤替代产业。着力培育石墨及石墨烯新材料、新能源产业，加快推进宝泰隆石墨烯和磷酸铁锂正极材料、华夏锂离子电池、宏达石墨综合加工项目，引进战略投资者有序推进石墨资源深度开发。加快奥瑞德蓝宝石二期项目建设，积极谋划引进三氧化二铝、钨钼材料等配套产业项目。加快再生资源系列项目建设进度，争取部分项目建成投产，通过国家“城市矿产”示范基地中期验收。整合农机装备制造业资源，以勃农兴达为龙头，完善农业播种、耕作、收获等系列农机具产品体系，做大做强省级农机产业园。立足木制品加工业集群发展，以双叶公司为牵动，积极引进小五金、外包装等上下游产业入驻木制品园区。培育发展健康医药产业，推进升华医疗器械项目建成投产，启动建设康草堂中药饮片、北斗生物人参加工等项目。做优煤炭循环经济。加快煤炭资源下游链条延伸拓展，推动煤化工向石油化工转变，发展精细化工、材料化工及合成化工。重点推进宝泰隆30 万吨稳定轻烃、5 万吨LNG 及隆鹏公司清洁化学品项目建设，支持北京三聚环保公司和隆鹏公司、鲁龙公司合作，启动建设泓泰兴和勃盛两个18 万吨合成氨30 万吨尿素项目。增强园区承载功能。探索推行产业园区“管委会+平台公司”运作模式，支持经济开发区以发酵产业为主攻方向，积极引进工业地产开发，推进标准化厂房及供电、污水处理、道路等基础设施建设，谋划建设生物发酵产业循环经济园区。盘活和清退园区内“僵尸企业”，提高园区土地利用率。推进再生资源园区一期基础设施收尾工程，开工建设供热中心、固废中心和污水处理厂，加快园区供热管网、道路建设。进一步扩大煤化工园区规模，搞好周边棚改腾空土地规划利用，完善园区水、电、路等基础设施。加速推进金沙健康产业园区建设，新增建设用地40 公顷，新建1 万平方米企业孵化器。提升招商引资实效。打好项目“储备之冬”会战，面向发达地区和产业转移重点地区，针对与我市产业发展方向高度契合的领域，谋划一批延伸产业链条、发展精深加工、产业配套协作项目，特别是成长性好、吸纳就业能力强的项目，深入开展驻点招商、挂职招商、专业招商、以企招商，设立政府性产业发展扶持基金，加大产业项目建设扶持力度，提高招商引资成功率。

　　（二）加快工业优化升级，增强经济增长支撑动力

　　依托现有产业基础和比较优势，通过改造提升、优化升级激发企业活力，切实增强工业经济综合实力。加快传统产业改造重组转型升级。推进宝泰隆120 万吨煤矿等矿井建设，加快煤矿实质性整合，在稳定原煤产量基础上，稳步淘汰洗煤和焦化落后产能。鼓励龙洋焦电等焦化企业引进合作伙伴，通过资本运营、股权合作、并购重组等方式，加快转型升级。采取减免缓等措施，切实减轻企业税费负担，针对生产经营困难企业，逐一会诊，分类处置，促进企业稳住生产，争取扭亏。“一企一策”帮助建成未投产项目及停产半停产企业搞好要素保障、市场开拓、产需对接，尽快投产复产。支持七矿公司解困发展。充分发挥七矿公司在人才、技术、装备和管理等方面优势，进一步加深市矿在安全监管、矿井改造、工程建设、物业管理等方面合作。支持七矿公司深化改革，盘活厂办、矿办企业以及利用废旧矿山、厂房等资产开发引进转型项目，推动企业主辅分离、分兵突围。帮助企业破解发展瓶颈。加快推进110KV 茄子河输变电工程及庆—鸡—林输变电工程建设，利用大唐电厂和部分企业自备电厂富余电量，以七矿公司电网为平台，争取用电大户和园区直供电政策支持，降低企业用电成本。探索建立新形式的中小企业融资担保体系，引导和鼓励金融机构加快金融创新，保障企业融资需求。引导企业树立资本运营意识，加快推动恒益电器、德彩光电等具备条件的企业“新三板”挂牌融资。用好定向减税、工业企业贷款周转金等扶持政策，支持中小企业技术改造和产品升级，鼓励大型企业加强与中小企业协作配套，促进中小企业加快成长，争取10 户以上企业进入规模上行列。

　　（三）深入调整农业结构，大力发展市场化农业

　　着眼市场需求，大力发展绿特色农业，加快农业向生态和有机方向转变，提高农业比较效益，不断增加农民收入。加快农业结构调整。在“绿”、“特”、“名”上下功夫，引导特色农业向多村一品、多乡一业发展，打造农业特产基地。以高钙菜、蓝靛果、黑甜甜葡萄、杂豆为突破口，以绿色有机为标准加大单品突破力度，特色作物种植面积达到30 万亩以上，绿色食品种植面积突破100 万亩。加大农田水利设施投入，推进勃利县节水增粮项目，建设生态高产标准农田40 万亩，新增水田4 万亩。大力发展林下经济，扩大平欧榛子、红松坚果林等种植面积，积极发展刺五加、五味子等北药种植以及木耳、大球盖菇等食用菌栽培。精准实施畜牧业贷款贴息和补贴，促进畜牧业规模化发展，扶持大兴牧业、五方种猪加快发展，大力发展肉羊养殖，完成简州大耳羊本地化驯养，尽快形成规模养殖。提升农业产业化水平。积极培育家庭农场、专业大户、农民合作社、产业化龙头企业等新型农业经营主体。扶持发展粮食、农畜产品、林产品深加工项目，抓好意晟园玉米、金穗米业水稻、东之星高钙菜、伊鑫清真食品、九龙泉蓝靛果酒、中利黄体素等深加工项目建设，发展壮大绿色食品产业。完善农业社会化服务体系。大力推进互联网+农业，加大“三品一标”创建力度，加强投入品监管，开工建设农产品检验检测中心，结合中国供销“绿网工程”，加强农产品质量追溯体系建设，省级以上龙头企业和“四星级”合作社及“三品一标”农产品全部实现可追溯。建立完善乡镇级良种繁育、技术推广、农资供应等综合性服务机构，健全仓储物流、冷藏保鲜、信息金融等服务与支持体系。集中打造“东北老勃”、“忍冬小子”、“三胜杂粮”等具有明显地域特征的农产品品牌，增强品牌影响力。

　　（四）提升三产层次水平，积极拓展经济增长空间

　　按照服务产业发展、扩大消费需求、完善城市功能的要求，不断拓展服务业发展空间，全面推进服务业发展提速、比重提高、水平提升。推动商贸物流产业加快发展。推进区域商贸流通一体化，完成中国供销商贸博览城项目一期、开工建设项目二期，推动鑫达源物流公路货运枢纽项目投入运行，启动黑龙江东部物流基地项目前期工作。引导传统商贸企业错位经营、跨界营销，推动线上线下结合，满足不同层次消费需求。增加荣盛达海关监管场所商检职能，为外贸进出口企业提供便利化服务，扶持宏泰松果、盛昌蔬菜、华璞木业等有基础、有潜力的企业不断开拓国外市场。促进电子商务和信息服务业提档升级。积极创建省级电商示范基地，充分发挥各电商产业园、创客空间的集聚和孵化作用，引导创业者和工商企业开展电子商务应用，进一步完善电子商务创意、设计、包装、融资、物流等软硬件设施，加快推进工商银行“融e 购”平台建设。积极引进全国知名电商，谋划实施蜂巢电子商务生态园项目，加深与阿里巴巴、京东等知名第三方电子商务平台合作，支持地方电商平台做大做强，率先在绿色食品、地方特产领域取得突破，电商交易额突破4 亿元。以信息惠民工程为牵动，结合“互联网+”三年行动计划，加强大数据管理信息平台建设，提升宽带网络和4G 网络覆盖面和服务水平，促进信息资源共享，加快城市智能化管理步伐。释放休闲养老、旅游等新型服务业潜力。加快推进万景集团石龙山国家森林公园项目建设进度，发展“候鸟式养老”产业；完善西大圈国家森林公园旅游道路、游客中心等设施建设，争创4A 级旅游景区。积极做好桃山湖国家湿地公园景区综合规划，谋划桃山湖整体开发。扩大居民服务性消费，加快发展健康养老、家政服务、休闲健身等服务业；提高生产性服务业水平，积极发展现代金融、商务咨询、服务外包、专业技术等现代服务业。

　　（五）扎实办好民生实事，不断增进人民群众福祉

　　越是经济困难时期，越要注重改善和保障民生，坚持雪中送炭，坚持持续发力，使改革发展成果更多惠及人民群众。加快保障性住房建设。完成煤矿棚改房后续分配安置工作，进一步完善如意家园配套公共服务设施。续建4421套采沉区棚户区保障房，新建1081 套，完成16607 套货币化安置。新建续建林苑之星417 套公租房，实施暖房子工程32万平方米。完善社会保障体系。更加关注低保、低收入群众等困难群体，加强低保及低收入群体帮扶及动态化管理，实施困难残疾人生活补贴和重度残疾人护理补贴制度。推进医疗保险“三保合一”，扩大新农合大病保险，启动城镇居民大病保险、参公事业单位工作人员和公务员工伤保险。加强特殊群体服务设施建设，推动市失能失智老人养护院医养结合示范院建设，完成社会福利院改扩建、残疾人康复中心项目，开工建设勃利县残疾人托养中心，搬迁市特殊教育学校。推动创业就业。落实好降低准入、先照后证、创业培训、融资担保、提高服务效率等优惠政策，激发群众创业创新活力和潜能。利用腾退办公楼和闲置厂房，建设小微企业创业园、科技企业孵化器，推动返乡农民工、大学生、复转军人、下岗失业职工以及有劳动能力残疾人等群体创业就业。鼓励居家创业就业，吸纳更多从业人员。支持七矿公司组织化分流富余人员，加强就业信息发布和就业岗位对接，通过公益岗位、煤矿托管、产业吸纳等途径，促进更多的人员分流就业。加强劳动监察联合执法，建立完善农民工工资保证金、应急周转金制度，保障农民工合法权益。促进教育均衡发展。完成职业技师学院幼儿教育实训中心、市一中艺体馆及运动场、岚峰中学教学楼项目建设，翻建扩建市第六小学、勃利新起小学教学楼，开工建设茄子河区青少年校外活动中心、吉兴中心小学、大四站镇中心幼儿园等教学楼及附属设施，茄子河区、勃利县义务教育均衡发展达到国家验收标准。提高卫生计生服务水平。抓好市人民医院整体升级改造和功能完善，推进规范化临床培养基地和传染病专科项目建设，完成勃利县人民医院内科楼、茄子河医院等续建工程。推动医疗资源向基层倾斜，完成红旗镇、杏树乡、青山乡、永恒乡4 个乡镇卫生院建设，新建茄子河区、新兴区疾控中心和新兴区新合社区卫生服务中心。推进卫生计生信息化升级改造，稳固提升医联体建设水平，推行双向转诊、“慢病”分级诊疗模式。加大疾病防控力度，健全卫生应急处置机制，全方位开展卫生计生专业技术人才培训。丰富群众文体生活。巩固短道速滑竞技体育优势，加强教练员队伍建设，创办短道速滑特色学校，积极开展“百万青少年上冰雪”活动。加强体育中心运营管理，承办更多体育赛事。深入开展群众文化活动，免费开放公益性场馆，开工建设市文化中心，改建完善电视发射塔发射台，继续完善社区、乡村等文化设施，新建12 个社区文化活动中心。加快农村人口脱贫步伐。全面实施精准扶贫、精准脱贫工程，逐步实现农村贫困人口全部脱贫。完成标准线以下贫困人口调查统计工作，建立扶贫台账，对贫困人口实行分类扶持。支持农民工返乡创业，培育新型职业农民，引导农民创业带动致富；巩固和完善农村社会保障体系，符合政策的贫困人口全部纳入低保范围，健全留守儿童、留守妇女、留守老人和残疾人关爱服务体系。按照先易后难，以促进基本公共服务均等化为重点，对口推进实施勃利县14个村“整村推进”扶贫开发。

　　（六）完善城乡基础设施，大力提升城市承载功能

　　加快推进新型城镇化建设，加强和改进城乡建设管理，全面启动采煤沉陷区综合治理工程，完善市政与公共服务设施，鼓励企业和市民通过各种方式参与城市建设、管理，实现城市共治共管、共建共享。抓好城镇基础设施建设。加快推进城市供水工程建设，确保隧洞主体工程贯通、管线工程竣工。完成净水厂工艺、设备、厂房更新改造及供水管网改造工程，实行工业用水和生活用水分供。实施雨污分流工程，建设雨水管网3.6 公里。推进原七矿供热区域老旧供热管网改造，新建续建供热管网9.5 公里。开工建设中燃公司天然气总站，完成北控集团与七矿煤气公司交接。加快推动牡佳客运专线前期工作，确保如期开工建设。完成城市西环路续建工程，推进建设大同街东延二期工程，改造阿尔乔姆路等老旧街路。新建北岸新城一级客运站，金沙客运站工程建成投入使用，更新新能源公交车辆40 台。提高城市管理水平。严格执行国家园林城市绿地规划标准，推动园林绿化改造升级，续建南出口绿化二期工程，新建学子园绿化项目，增加小区庭院、公园广场、城市街路等点式绿化景观。加强路灯盲点区域建设，主城区背街巷路、居民小区等实现全覆盖。进一步理顺弃管楼管理体制，建立市区两级小区物业管理应急基金，解决困扰居民生活难题。坚持“六化一考”机制，完善城市精细化管理，加强商圈周边、背街巷路、城乡结合部环境整治和住宅小区公共空间管理，做好冬季清冰雪工作。不断改善环境质量。继续推进“清洁空气行动计划”，整治燃煤污染、工业污染和生活污染，淘汰全部到期黄标车。制定并积极推进“水污染防治行动计划”，加强水源地保护，实行最严格水资源管理制度。加大对环境违法行为的打击力度，开展清理整顿环保违规建设项目专项行动，全面推行环境监管网格化管理。积极推进主要污染物减排工作，确保完成年度污染物总量控制指标。启动市节能监察中心，加强对重点用能单位节能执法监察，万元GDP 能耗下降4%。推进美丽乡村建设。新建农村饮水安全工程16 处，改造50处。抓好乡村路网建设，推进万建路、七密路等9 条农村公路改造。加强乡村环境整治，实施村内街路硬化、绿化、亮化，做好垃圾清运和街道保洁。整治农业污染，引导和鼓励农民减少农药、化肥、除草剂施用，推进秸秆等种养业废弃物资源化利用、无害化处置。

　　（七）突出抓好安全生产，全力维护社会和谐稳定

　　牢固树立安全发展理念，不断改进社会治理方式，全面落实各行业各领域安全责任，确保社会安全稳定。抓好煤矿安全生产。继续加强煤矿分类监管和井下24 小时旁站式监管，加强瓦斯、水患等重点环节管理，持续开展“打非治违”等专项行动，加强煤管、安监、公安、监察、供电等部门综合执法，坚决遏制和严厉打击煤矿违法违规开采行为。推进煤矿整顿关闭工作，做好煤矿企业整治整合、兼并重组、证照办理、安全托管、改造升级等后续工作。提升安全基础水平。加强各行业各领域安全体系建设，继续实施安全责任区及安全班组建设，探索推动乡镇级安全综合管理。启动黑龙江省东部煤化工应急救援基地一期工程建设，提升应急救援能力。开展食品安全城创建，完善食品药品检验检测体系，完成市检验检测与认证中心项目建设。强化各行业各领域安全隐患大排查大整治，确保危险化学品、食品药品、消防、人员密集场所、道路交通等安全。加强社会综合治理。深入推进“平安七台河”建设，积极构建立体化社会治安防控体系，完善“平安家园”和居民小区视频监控系统补点建设，深化城乡社区警务工作，实施“五位一体”巡逻联动执勤模式，提高街面见警率，保持对违法犯罪的高压态势。强化社区矫正工作，严密重点人员管控，全面加强寄递物流、危爆物品安全管理，突出抓好群体性事件的预防和处置。维护社会和谐稳定。大力推行网上信访，畅通信访渠道，完善社会矛盾预警调处化解机制，及时化解社会矛盾。坚持市级领导定期接访、疑难信访问题综合评议制度，推动合理诉求依法解决；落实诉访分离制度，依法惩治信访活动中违法行为。加快诚信体系建设步伐，严厉打击违约失信行为。

　　（八）始终坚持问题导向，全面深化体制机制改革

　　落实好中央、省市委各项改革举措，加大重点领域和关键环节改革推进力度，进一步释放发展活力。深化经济体制改革。推进财税体制改革，完善市、区级财税分配机制，保证事权财权相匹配。落实国税、地税征管体制改革。做好政府债务置换工作，规范政府性债务管理体制。构建多元化融资体系，盘活政府优良资产，推动财政资金与社会资本融合。鼓励社会资本参与市政设施、公共服务等项目建设，推进政府向社会力量购买公共服务改革。继续推进招投标体制改革，整合建立公共资源交易平台。推进农业综合改革。加快农村土地确权改革，推进84 个行政村土地确权工作。完成红光村、东胜村和万宝村农村集体产权制度改革试点。规范农业专业合作社建设，引导合作社向“联合社”发展，鼓励农民带地入社。建立土地流转交易平台，推动土地适度规模经营。探索扩大农村土地经营权抵押、担保贷款，开展多种形式的农业保险和互助合作保险，实现农村基础金融服务全覆盖。完善文教卫生体制改革。全面推进公立医院综合改革，探索建立符合医疗行业特点的人事薪酬制度，建立精细化管理和考核评价机制。启动新农合支付方式改革。统筹义务教育人才资源均衡配置，开展校长、教师交流改革试点，继续推进初中升学考试改革和综合素质评价改革。抓好其他领域改革。推进事业单位分类改革和厂办大集体改革，稳妥实施县以下机关公务员职务与职级并行制度，全面推行机关单位公务用车改革。深化户籍制度改革，推行居住证制度，全面放开落户限制，解决无户口人员落户问题。深入推进生态文明体制机制改革，适时启动生态保护红线划定工作，优化发展空间布局，确定生态环境安全底线。有序推进国有林场改革，完善国有森林资源资产管理制度。

　　三、切实加强政府自身建设

　　以增强政府执行力和公信力为重点，进一步转变政府职能，努力建设人民满意政府。

　　强化担当意识，打造责任政府。深入践行“三严三实”，坚持不懈纠正“四风”，持续开展“庸懒散浮”专项治理，加大对基层单位工作人员吃拿卡要、不作为、慢作为、乱作为的整治力度，突出解决好“不一次告知”、“门好进、脸好看，事还是难办”、“下梗阻”等问题。进一步优化发展环境，坚持零距离接触、零关系办事、零利益服务、零障碍入驻“四个零”工作机制，全力服务项目引进和企业发展。强化企业投诉服务中心作用，严厉查处破坏发展环境典型案件。认真落实“宁静工作日”制度，严禁随意入企检查，维护企业正常生产经营秩序。继续弘扬求真务实、敢于担当的工作作风，以踏石留印、抓铁有痕的劲头推动各项工作落实。

　　强化法治意识，打造法治政府。积极推进依法行政，完善重大行政决策风险评估、专家咨询论证、公众参与、集体决策、后评估以及监督检查制度，严格执行规范性文件备案审查制度，推动行政决策民主化、制度化、科学化。深化行政执法体制改革，严格规范行政执法自由裁量权，加快推进综合执法，全面落实行政执法责任制，杜绝执法不严不公现象。全面推行政府法律顾问制度，发挥法律顾问在各级政府和部门重大行政决策中的合法性监督作用，确保行政行为合法性。

　　强化效率意识，打造服务政府。进一步推进简政放权，加快行政审批制度改革，清理和规范“中介服务”机构，认真开展降低实体经济企业成本行动。进一步落实利民便民要求，简化行政公共服务流程，砍掉一切无法律依据的无谓证明和繁琐手续。建立投资项目在线审批监管平台，全面落实重点产业项目领办代办制，提供全过程全方位服务。深入推进“企事厂办”，严格落实窗口部门“无间断”办事制度，推动网上政务服务中心高效运转，全力提升政府行政效率和服务水平。

　　强化廉洁意识，打造阳光政府。认真落实党风廉政建设主体责任，做好《中国共产党纪律处分条例》和《中国共产党廉洁自律准则》的贯彻落实，全面加强政府系统廉政建设，对违法违纪和腐败行为“零容忍”，坚决予以查处。加强公共资金、国有资产资源监管，推进审计监督全覆盖。改革政府采购监管与操作机制，政府性资金项目全部纳入政府采购范畴。加大政务公开力度，保障群众的参与权、知情权和监督权。落实好政府权力清单、责任清单，规范权力使用和运行程序。认真接受人大的法律监督和工作监督，自觉接受政协民主监督，积极回应群众呼声和社会关切，进一步树立为民务实清廉的政府形象。

　　各位代表，七台河转型发展任重而道远，我们肩上的责任和使命重于泰山。新的一年，让我们在省委省政府和市委的坚强领导下，坚定信心，攻坚克难，狠抓落实，全力做好“十三五”开局之年各项工作，为七台河全面建成小康社会而努力奋斗！
